# Methylphenidate improves motor functions in children diagnosed with Hyperkinetic Disorder

**DOI:** 10.1186/1744-9081-5-21

**Published:** 2009-05-13

**Authors:** Liv Larsen Stray, Torstein Stray, Synnøve Iversen, Anne Ruud, Bjørn Ellertsen

**Affiliations:** 1 Department of Child and Adolescent Health, Sørlandet Hospital, Norway; 2 The Reading Centre, Faculty of Education and Arts, University of Stavanger, Norway

## Abstract

**Background:**

A previous study showed that a high percentage of children diagnosed with Hyperkinetic Disorder (HKD) displayed a consistent pattern of motor function problems. The purpose of this study was to investigate the effect of methylphenidate (MPH) on such motor performance in children with HKD

**Methods:**

25 drug-naïve boys, aged 8–12 yr with a HKD-F90.0 diagnosis, were randomly assigned into two groups within a double blind cross-over design, and tested with a motor assessment instrument, during MPH and placebo conditions.

**Results:**

The percentage of MFNU scores in the sample indicating 'severe motor problems' ranged from 44–84%, typically over 60%. Highly significant improvements in motor performance were observed with MPH compared to baseline ratings on all the 17 subtests of the MFNU 1–2 hr after administration of MPH. There were no significant placebo effects. The motor improvement was consistent with improvement of clinical symptoms.

**Conclusion:**

The study confirmed our prior clinical observations showing that children with ADHD typically demonstrate marked improvements of motor functions after a single dose of 10 mg MPH. The most pronounced positive MPH response was seen in subtests measuring either neuromotor inhibition, or heightened muscular tone in the gross movement muscles involved in maintaining the alignment and balance of the body. Introduction of MPH generally led to improved balance and a generally more coordinated and controlled body movement.

## Background

In a previous study we found that a high percentage of children diagnosed with HKD showed functional problems associated with motor inhibition and stability problems[1]. Another study showed that children with ADHD, who responded well to central stimulant medication on attention and hyperactivity problems, showed more motor problems than those who did not respond to this medication[2].

The purpose of this study was to investigate the effects of methylphenidate (MPH) on motor function in children with Hyperkinetic Disorder, HKD/F90.0 [3]. HKD is quite similar to Attention Deficit Hyperactivity Disorder, combined subtype, ADHD-C [4]. Motor function was assessed using the Norwegian version of Motor Function Neurological Assessment battery, MFNU [5].

For further discussion of diagnostic considerations, see Ellertsen and Johnsen [6]. In a previous paper [1] addressing motor problems in boys with ADHD-C/HKD, compared to a control group without ADHD, it was found that a high percentage of the ADHD group showed motor problems. The percentage of 'severe problems' within the 17 sub tests of MFNU ranged from 44 – 84%, typically over 60%. In all, 80–96% of the subjects showed problems when the scoring categories 'moderate'and 'severe' problems' were combined. The rate of motor problems reported in our study was markedly higher than the 30 – 50% typically reported in the literature [7-10].

Diagnosis of ADHD/HKD is made on the basis of behavioural criteria alone. It involves a deficit in sustained attention, impulse control and activity regulation across situations like at home and at school for at least the past 6 months, starting before the age of 7 years [4,11]. There is general agreement that ADHD is primarily neurobiologically based [12,13]. Neurophysiological studies have shown that certain brain areas closely associated with motor control seem to be involved in deficits in motor inhibition, particularly the frontostriatal system, basal ganglia and cerebellar vermis [14,15].

Methylphenidate (MPH) and d-amphetamine are considered the treatment of choice for ADHD, with a number of studies indicating that approximately 80% of subjects with ADHD show clinically significant benefits from such treatment [16,17]. MPH is believed to activate self-regulatory control processes, thereby affecting what is believed to be the core neurofunctional problems of the condition and improving motor functions [18]. Thus, Lerer et al. [19] showed that administration of MPH improved handwriting in children with what they called Minimal Brain Dysfunction (MBD). This term has been abandoned, but it is quite reasonable to assume that what is now termed ADHD was a significant part of it [6]. In children with the combined DSM IV diagnosis of ADHD and Developmental coordination disorder (DCD), Flapper et al. [20] showed that medication with MPH for 5 weeks improved handwriting and manual dexterity. Tucha and Lange [21-23] concluded that children with ADHD, when withdrawn from medication with MPH, showed poorer handwriting legibility and accuracy in comparison with the control group. Improvement of qualitative aspects of handwriting was found following treatment with stimulant medication. Zeiner et al. [24] found that boys diagnosed with ADHD displayed a significantly lower counter score on the Maze Coordination Test during MPH medication compared to placebo. The testing was performed on the 15th or 16th day of each medication period. Rubia et. al. [25] demonstrated that MPH improved motor timing in children with ADHD. MPH also improved speed of inhibition and response execution processes [26]. O'Driscoll et al. [27], assessing eye movements in children with ADHD, found that MPH improved motor planning and response inhibition. Rubia et al[25] found that prolonged administration, but not a single dose, of methylphenidate reduced the variability of sensorimotor synchronization and anticipation.

In the process of developing the MFNU, Stray [2] repeatedly observed that not only a prolonged treatment, but also a single dose of central stimulants improved motor performance and attention in individuals with ADHD. This effect was usually observed 1/2 – 1 hour after medication, rapidly subsiding after 3–4 hr when a dosage of 10 mg MPH was delivered. It was also repeatedly observed that when the same children were retested without medication, the motor problems had reoccurred. This pattern was seen in children of both sexes, and over a wide age range, including children who had been medicated for years. The positive effect of MPH on motor performance was observed not only on the MFNU, but in many areas of daily living as well.

A pilot study, assessing 6 ADHD children with MFNU, showed that all children had motor problems and that administration of 10 mg MPH gave positive effects on motor performance in all subjects after 90–120 min [2]. In order to pursue these findings, the present double-blind MPH/placebo study was carried out, using the MFNU as a test-retest procedure on 24 drug naïve boys with the ADHD-C/HKD diagnosis.

Our hypothesis was that a single dose of MPH significantly would improve MFNU motor function in children with ADHD-C/HKD, compared to performance without medication.

## Method

### Participants

A total of 25 out-patients boys, aged 8–12 years (mean age 10.2, SD 1.3) who were recently diagnosed at the Child Psychiatry Department of a regional hospital were recruited for the study. The diagnosis of ADHD was made according to the ICD-10 criteria for Hyperkinetic disorder (HKD) F90.0 [11]. They were all candidates for methylphenidate evaluation at the hospital, and had no prior experience with stimulant medication. The diagnosis was set as a part of the ordinary assessment routines, based on clinical interviews and consultations by a physician or a clinical psychologist who incorporated information from the sources available. These included a report from a parent or guardian, school reports for the last 12 months, reports from other health professionals, and behavioural observations during the assessment period. Parents and teachers of the children completed standardized questionnaires (a Norwegian version of Barkley's DSM-IV rating scale for ADHD)[28]. The WISC-R was administered in all cases. Mean IQ was 97.6 (SD 15.9). Children were excluded if they met ICD-10 criteria for Hyperkinetic disorder associated with conduct disorder (F 90.1), a depressive or anxiety disorder, Asperger or Tourette syndrome or known epilepsy. At a later point in time, and independent of the present study, 23 of the children were assessed for MPH effect on the core symptoms of ADHD at home and at school. The remaining 2 children were, due to the parent's choice, withdrawn from further MPH assessments at the hospital. 21 of the 23 subjects responded positively to MPH.

### Motor assessment

The MFNU used in this study is thoroughly described in a user manual and an accompanying DVD with videos of MFNU-assessments of children without ADHD, and children with ADHD tested without and with MPH medication [5]. The MFNU was developed over a 10 year period in the 1990'ies in close collaboration with well-educated and specialized personnel trained within the field of ADHD, learning and conduct problems. Some of the subtests were developed as part of the MFNU, and some are modified versions of subtests from the Danish 'Funksjonsnevrologisk Undersøgelse' (FNU)[29]. The subtests were primarily chosen and designed to reveal problems with motor inhibition and increased muscle tone, not motor problems in general. A qualitatively based scoring system is used. The test is performed in a highly "dynamic" and interactional way with no limits concerning time and number of attempts, in order to focus the attention of the child [2,5].

Most of the subtests of the MFNU are performance tests where the child is given an instruction to perform a certain task (subtests 01–12). Subtests 13–16 are passive tasks where the tester evaluates muscular resistance while assessing hips and feet. Item 17 'Synkinesis', is an evaluation of the presence of synkinetic movements during the examination. Subtest 18, 'Palpation', was omitted from the study, as it can not be scored on the basis of video recordings. Table 1 presents a brief description of the subtests used.

**Table 1:** The subtests of MFNU used in the study

Name of subtests and video examples	Description
01. Dynamic balance-2 legs	Three sideway jumps within marked squares, back and forth. The entire process is repeated three times without stopping.
02. Dynamic balance-1 leg	Three sideway jumps on one leg within marked squares, back and forth. The entire process is repeated three times without stopping. Both legs are tested.
03. Diadochokinesis-right 04. Diadochokinesis-left	Pronation-supination of one hand, the elbow flexed 90 degrees. The hand is held as an "extension" of the lower arm. The exercise is performed for approximately 15–20 seconds.
05. Reciprocal coordination	Alternate clenching of one fist, and stretching of the other in a rhythmic manner, for about 15 seconds. Fingers should be nearly completely extended after the hand has been clenched. Elbows at a 90 degree angle, palms facing upwards.
06. Thumb movement	The tip of the other fingers are successively touched with the palmar surface of the tip of the thumb. After each opposition the child extends and abducts the thumb. Both hands are tested for approximately 20 seconds.
07. Throw ball	The tester plays ball with the child. A fearly large ball is used. The child has to throw with dominant arm in an upwards position. Shoulder movement is scored.
08. Catch ball	The tester plays ball with the child. A tennis ball is used. The child has to catch the ball with one hand, fingers flexed, without touching the body.
09. Walking	Walking with toes alternately pointing outwards ("Chaplin") and inwards, followed by walking on the outer foot rend (Fog's test) and inner foot rend.
10. Lifting arm	Lies prone, arms in a 45 degree angle from midline, lifting one arm with the palm of the hand facing the floor.
11. Lifting leg	Lies prone, spina iliaca anterior is touching the floor while lifting one stretched leg at a time.
12. "Flying"	Lies prone, the arm in a 45 degree angle from midline, lifting head, arms and legs.
13. Passive abduction-right hip 14. Passive abduction- left hip	Lies supine. Tester holds the child's knee and hip in a flexed position. The tester stretches and flexes the leg to elicit a relaxation of the hip muscles, and abducts the leg. The sides are evaluated separately.
15. Passive movement-right foot 16. Passive movement-left foot	Lies supine. Tester examines passive movement with dorsal flexion and eversion/plantar flexion of the right and the left foot.
17. Synkinesis	'Synkinesis' is not a separate test, but an item for the evaluation of synkinetic movements registered in one or more subtests. When observed, the tester tries to correct it. The remaining synkinesis after correction is scored.

The MFNU is scored on a sheet applying a ranked 3-category format (0–1–2). The subtests are scored according to the criteria presented in Table 2. More detailed criteria for each subtest are given in the MFNU manual and visualized in the accompanying DVD [5]. Inter-tester reliability analyses have shown high to very high rater agreement on all subtests (Cohen's Kappa ranging from .67 to 1.00) [2].

**Table 2:** Scoring criteria for the 17 subtests of MFNU

	Score:	Criteria		
		subtests 01–12	subtests 13–16	subtest 17
0	'No problems'	The task is performed with no problems and little effort	Normal resistance against the movement is registered	Only sporadic synkinetic movements are registered
1	'Moderate problems'	The task is performed according to instruction, but with lot of attention and effort, or quality of performance is below what is expected for age	Resistance against the movement is registered	Moderate synkinetic movements are registered in one or more subtest
2	'Severe problems'	The child can not perform the task according to the instruction	Severe resistance against the movement is registered	Pronounced synkinetic movements are registered in one or more subtest

A scoring system based on observed *changes *in performance is used when comparing test-retest performances. A change score ranging form -3 to +3 is applied, where a score of '0' means no change. A score of '+1' or '-1' means that observable change in performance is registered, but not qualifying for a score across a category. This scoring system permits a more subtle evaluation of change in performance than if the 3-category system had been used for this purpose. Intraclass correlation between two trained raters ranged from .79 to .99 (Stray et al. article in preparation). See Table 3 for a detailed description of the scoring system. See also the MFNU manual for specific scoring criteria, and the accompanying DVD for video illustrations of the dual display comparison involved in the test-retest rating procedure.

**Table 3:** The change scores used in MFNU in test-retest procedures

	Change score
Neg. change across two categories	-3
Neg. change across one category	-2
Neg. change within same category	-1
No change	0
Pos. change within same category	1
Pos. change across one category	2
Pos. change across two categories	3

### Design

A double-blind, placebo controlled, crossover design was applied, using study capsules with a single dose of 10 mg immediately released methylphenidate (MPH) or placebo (approved by the Norwegian Medicines Control Authority). The children were randomly assigned into two groups by a simple randomization procedure, resulting in 15 subjects allocated to Group A (receiving placebo on day 1 and methylphenidate (MPH) on day 2) and 10 subjects to Group B (receiving MPH on day 1 and placebo on day 2). The researchers, the participants and the rater of the videos were blinded with the regard to group assignment. The "key" to the treatment assignments was unavailable throughout the study period. One patient belonging to group B dropped out after the first day, reducing this group to 9 subjects.

### Procedures

The children were tested individually with the MFNU two times a day on two different days, with an interval of 2 to 24 days (median 5 days). Study capsules were administered according to the design displayed in Table 4. The 10 mg of MPH, or placebo, was administered immediately after the baseline trial each day (Trial 1 and 3 respectively) (the result of the two trials without medication were presented in a previous study, where the ADHD group was compared to a control- group [1]). The retest was done 90 min after administration of MPH/placebo both days.

**Table 4:** The cross-over design of the experiment

	Day 1		Day 2	
	Trial 1	Trial 2	Trial 3	Trial 4
Group A (n = 15)	No medication	Placebo	No medication	10 mg MPH
Group B (n = 10)*	No medication	10 mg MPH	No medication	Placebo

* 1 of the subject of Group B did not attend Day 2, reducing the N to 9.

The children were assessed individually at the hospital with their parents being present. All sessions were videotaped. Prior to the test sessions all subjects went through a consultation with a physician (the fourth author), and a preliminary MFNU assessment with the physical therapist (the first author). The preliminary trial was given in order to avoid possible negative test results due to distraction or to emotional reaction to an unfamiliar situation.

The rating of each child was performed by a trained physiotherapist at a later point in time, based on the videotapes from each session. The rater had no prior knowledge to any of the children. The sessions were displayed in blinded order on two parallel screens showing the baseline trial (Trial 1 or 3) on the left screen and the compared MPH/placebo (Trial 2 or 4) on the right. The sound was turned off during the rating in order to ensure that verbal comments from the participants would not influence the rater.

The scoring sheet consisted of one row and three scoring columns for each subtest, representing the scoring categories 0, 1 and 2. The baseline scores were evaluated and marked first, using a pencil with a specific colour. The scores of the comparison trials were subsequently marked within the same set of columns, with different colours for each trial.

Each subtest was given a Category score from 0–2 on each trial. In addition a separate Change score was set for each of the successive trial according to the rules described in Table 3. A 'Total score', which is the sum of the Category scores of 17 subtests for each subject, was computed for each of the four trials. The Total score ranges from 0 to 34. In a previous study, a very high internal consistency within the total set of sub-tests was found (Cronbach's Alpha = 0.98) [1]. This variable is assumed to be continuous and was used to illustrate the severity of the motor problems.

### Data analyses

The statistical analyses were carried out using the SPSS version 15.0. Distributions of the scoring categories (0–1–2) and of the change scores for each subtest, and for the Total score were obtained from the registered baseline, placebo and MPH data. Effects of MPH were analysed using the Wilcoxon Signed-rank test for related samples to compare the performances during baseline, placebo and MPH trials on each of the 17 subtests and on the Total score. Mann Whitney U-tests were used to compare the two groups. Non-parametric tests were applied because the data were not normally distributed, and because the measurement level was ordinal for the subtests. Cohen's *d*-analysis was used for calculation of the effect size of the Total score.

### Approvals

The study was approved by the Norwegian Data Inspectorate, The Regional Committee for Medical Research Ethics, Norwegian Medicines Agency, the research committee and the director of medicine at the Department of child and adolescent mental health, Sørlandet Hospital, Kristiansand, Norway,

## Results

When comparing the placebo performance of the A and B groups on the individual subtests no significant differences were found for 16 of the 17 subtests (Mann-Whitney U-test). A tendency towards a weaker placebo performance on some of the subtest was registered in the group receiving placebo on the second day (fourth session). The differences were non-significant for the subtests except 'Thumb movement' (p = .04). There were no significant differences between Group A and Group B on Total score when comparing the baseline, MPH or placebo sessions. This indicated that the A and B-group could be treated as one group (N = 24) in further analyses.

Table 5 shows the relative distribution of scores in the categories 0–2 for the whole group on each subtest on the baseline, MPH and placebo condition. The most pronounced improvements with MPH compared to baseline were observed on the passive movement tests (13–16), the extension subtests (10–12) and 'Throw ball' (07). The movement of the hips was markedly better for a majority of the children (83.3% score 2 on baseline 1 and baseline 2 and 12.5% on the MPH trial for the right hip and a change from 79.2% to 8.3% for the left). On the MPH trial for the subtest 'Lifting arms' (10), many of the children were able to lift one arm at a time with the palm of the hand facing the floor, maintaining the angle from midline. Only 25% compared to 58.3% on the baseline trials showed severe problems on this item. When medicated with MPH, only 16.7% of the children showed severe problems on the subtest 'Lifting legs' (11), and 33.3% of the children were able to lift one stretched leg at a time keeping the spina iliaca on the floor, compared to 4.2% on baseline 2 and the placebo trial. When performing the subtest 'Throw ball' (07) with MPH, only 16.7% of the children, as compared to 58.3% on the baseline 1 and placebo trial, showed severe problems when throwing with the arm in an upward position. Passive movement of the right foot was improved by MPH (70.8% with severe problems on baseline 1, 79.9% on baseline 2 and only 4.2% on the MPH trial).

**Table 5:** The percent distribution of the 3 scoring categories on baseline 1, baseline 2 (trial 3), MPH and placebo trial (N = 24)

	Score 0 %				Score 1 %				Score 2 %			
Subtests of MFNU	Bas.1	Bas. 2	MPH	Plac.	Bas.1	Bas. 2	MPH	Plac	Bas. 1	Bas. 2	MPH	Plac.
01. Dynamic balance, 2 legs	12.5	12.5	20.8	12.5	33.3	33.3	41.7	29.2	54.2	54.2	37.5	58.3
02. Dynamic balance, 1 leg	4.2	0.0	16.7	0.0	25.0	29.2	37.5	25.0	70.8	70.8	45.8	75.0
03. Diadochokinesis, right	8.3	4.2	12.5	4.2	25.0	25.0	54.2	25.0	66.7	70.8	33.3	70.8
04. Diadochokinesis, left	4.2	0.0	8.3	0.0	29.2	9.2	45.8	29.2	66.7	70.8	45.8	70.8
05. Reciprocal coordination	8.3	0.0	20.8	0.0	29.2	33.3	54.2	33.3	62.5	66.7	25.0	66.7
06. Thumb movement	12.5	12.5	29.2	12.5	20.8	20.8	45.8	25.0	66.7	66.7	25.0	62.5
07. Throw ball	8.3	8.3	25.0	4.2	33.3	37.5	58.3	37.5	58.3	54.2	16.7	58.3
08. Catch ball	16.7	12.5	20.8	8.3	33.3	37.5	54.2	41.7	50.0	50.0	25.0	50.0
09. Walking	20.8	16.7	20.8	8.3	12.5	12.5	41.7	12.5	70.8	70.8	37.5	70.8
10. Lifting arm	8.3	0.0	33.3	0.0	33.3	41.7	41.7	33.3	58.3	58.3	25.0	66.7
11. Lifting leg	12.5	4.2	33.3	4.2	41.7	45.8	50.0	45.8	45.8	50.0	16.7	59.0
12. "Flying"	8.3	0.0	41.7	0.0	29.2	37.5	37.5	37.5	62.5	62.5	20.8	62.5
13. Passive abduct. right hip	12.5	4.2	62.5	8.3	4.2	12.5	25.0	8.3	83.3	83.3	12.5	83.3
14. Passive abduct. left hip	8.3	12.5	75.0	4.2	12.5	8.3	16.7	16.7	79.2	79.2	8.3	79.2
15. Passive move. right foot	8.3	4.2	37.5	4.2	20.8	16.7	58.3	16.7	70.8	79.2	4.2	79.2
16. Passive move. left foot	16.7	8.3	62.5	4.2	20.8	33.3	33.3	33.3	62.5	58.3	4.2	62.5
17. Synkinesis	12.5	4.2	12.5	4.2	20.8	29.2	62.5	29.2	66.7	66.7	25.0	66.7

Score 0:'No problems'

Score 1:''Moderate problems'

Score 2:'Severe problems'

The Wilcoxon test showed significant improvements on all subtests when comparing baseline 1 to the MPH trial (see Table 6). No significant differences were found between the baseline 1 and the placebo trial except for subtest 10 'Lifting arm' which showed more problems on the placebo trial (p < .05).

**Table 6:** Results of significance tests (Wilcoxon signed ranks test) when comparing MPH/placebo scores against baseline 1(category scores).

	MPH trial – baseline1		placebo trial – baseline1	
Subtets of MFNU	p-value	z	p-value	z
01. Dynamic balance, 2 legs	.014	-2.449^a^	.317	-1.000^b^
02. Dynamic balance, 1 leg	.007	-2.714^a^	.317	-1.000^b^
03. Diadochokinesis, right	.003	-3.000^a^	.157	-1.414^b^
04. Diadochokinesis, left	.014	-2.449^a^	.157	-1.414^b^
05. Reciprocal coordination	.003	-2.972^a^	.083	-1.732^b^
06. Thumb movement	.004	-2.889^a^	.317	-1.000^b^
07. Throw ball	.000	-3.500^a^	.317	-1.000^b^
08. Catch ball	.008	-2.646^a^	.317	-1.000^b^
09. Walking	.005	-2.828^a^	.317	-1.000^b^
10. Lifting arm	.000	-3.500^a^	.046	-2.000^b^
11. Lifting leg	.001	-3.207^a^	.083	-1.732^b^
12. "Flying"	.000	-3.626^a^	.157	-1.414^b^
13. Passive abduct. right hip	.000	-3.852^a^	.317	-1.000^b^
14. Passive abduct. left hip	.000	-4.072^a^	.317	-1.000^b^
15. Passive move. right foot	.000	-3.906^a^	.083	-1.732^b^
16. Passive move. left foot	.000	-3.729^a^	.180	-1.342^b^
17. Synkinesis	.002	-3.126^a^	.157	-1.414^b^

a. Based on positive ranks

b. Based on negative ranks

**Figure 1:**
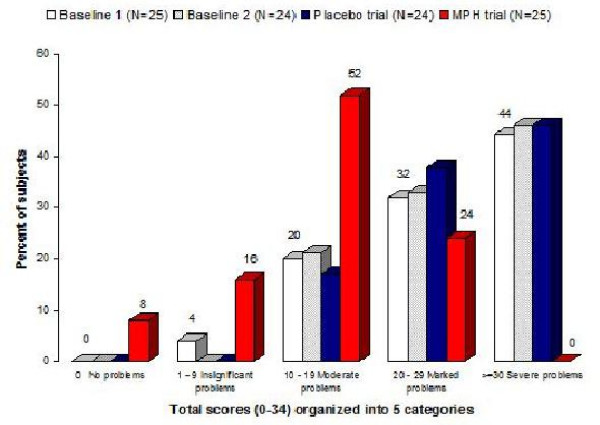
**The Total score for the baseline, placebo and MPH trials (N = 25)**. shows the distribution of the Total score for the baseline, placebo and MPH trials (N = 25). The Total score is categorized into 5 categories, ranging from 0 to 34, where a score of 0 means 'no problems' on any subtest, and 34 means a score of 2 ('severe problems') on all 17 subtests.

Fig. 1 presents the distribution of the 'Total Problem Score' (N = 24) grouped into 5 categories under the 4 different testing conditions. With MPH only 24% of the subjects showed marked or severe problems (Total score 20 or higher), as compared to 76% on the baseline trial and 84% on the placebo trial. The figure also clearly reveals that placebo had no positive effect on performance.

**Figure 2:**
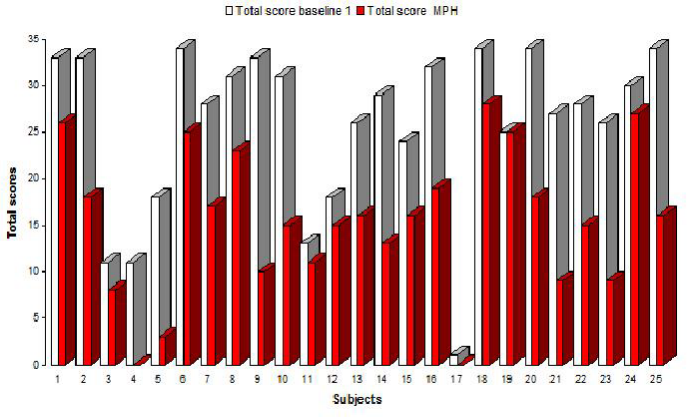
**Total score for the baseline 1 and MPH trials for 25 subjects**. shows the Total scores on the baseline 1 and the MPH trials for each subject.

Fig. 2 illustrates the Total score differences between baseline 1 and MPH and between baseline 1 and placebo performances for each subject. Subject no 19 showed no effect of MPH.

**Figure 3:**
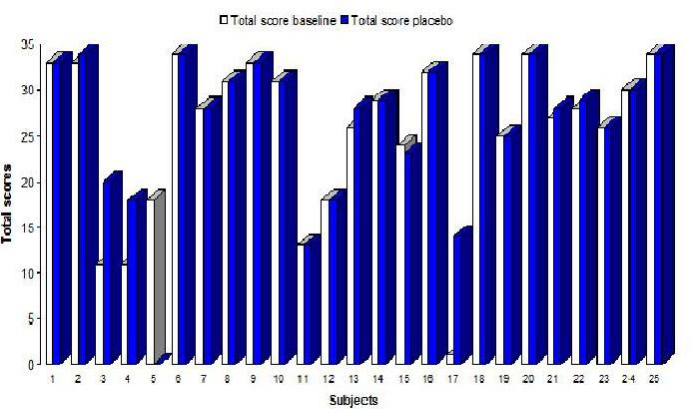
**The Total scores for each subject on the baseline 1 (N = 25) and placebo trials (N = 24)**. shows the Total score on the baseline 1 and the placebo trials for each subject. Subject nr 5 did not attend the placebo trial.

A marked negative responses to placebo compared to baseline 1 was registered in 3 subjects (Fig. 3). Only minor changes were registered from baseline 1 to placebo for the rest of the sample indicating that the placebo condition yielded no positive effect on motor performance.

When the 7 category 'Change score' system was applied, the passive movement subtests (13–16) showed the highest percentage of positive change across two categories (from 'severe' to 'no problems') from baseline to MPH trial (See Table 7). On the dynamic balance subtests (01–02) 'Diadochokinesis, left', (04) 'Reciprocal coordination' (05) and 'Walking' (09) a relatively high percentage of the subjects were scored in the +1 category ('Positive change within the same category').

**Table 7:** Distribution of Change scores on 17 subtests of MFNU when performance on baseline and MPH trials are compared (N = 25)

	Negative change one category	Negative change within same category	No change	Positive change within same category	Positive change one category	Positive change two categories
Subtests of MFNU	Score = -2	Score = -1	Score = 0	Score = +1	Score = +2	Score = +3

01. Dynamic balance, 2 legs	0	1	6	12	6	0
02. Dynamic balance, 1 leg	0	0	6	11	7	1
03. Diadochokinesis, right	0	0	7	10	8	0
04. Diadochokinesis, left	0	0	6	11	8	0
05. Reciprocal coordination	0	0	5	12	6	2
06. Thumb movement	0	0	5	8	8	4
07. Throw ball	0	0	6	8	11	0
08. Catch ball	0	0	6	12	7	0
09. Walking	0	1	5	9	10	0
10. Lifting arm	0	0	3	10	11	1
11. Lifting leg	0	0	3	10	11	1
12. "Flying"	0	0	3	6	13	3
13. Passive abduct. right hip	0	0	5	1	7	12
14. Passive abduct. left hip	0	0	4	1	6	14
15. Passive move. right foot	0	0	4	1	14	6
16. Passive move. left foot	0	0	6	1	11	7
17. Synkinesis	1	1	5	8	10	0

During a later assessment performed by the hospital 21 out of the 23 boys showed a positive MPH response at home and at school on the core symptoms of ADHD. One of the two non-responders had a Total score of 18 on the baseline and placebo trials and of 15 on the MPH trial. The other subject obtained a Total score of 23 on baseline and placebo trials, and 16 on the MPH trial (improvement on the 'passive movement tests', 'Lift arm' and 'Synkinesis').

A Cohen's *d*-analysis of effect size was performed applying the Total score of the MPH and the baseline 1 trials to establish the statistical power of the effects shown in the study. A Cohen's *d *of 1,27 was found, which according to Cohen [30] is a substantial effect size.

## Discussion

The results support our hypothesis that MPH would significantly improve motor function in children with ADHD-C/HKD compared to performance without medication. Improved performance with MPH was demonstrated on all the 17 subtests of the MFNU. Performance under placebo conditions was not significantly different from performance during the baseline trials ruling out that the improvements on MPH might be explained by a placebo effect.

While improved performance with MPH was registered on all the subtests of the MFNU, the greatest improvements were observed on subtests constructed to measure increased muscle tone in the "movement muscles" (for instance m. Latissimus dorsi and m. Psoas major) (subtests 07, 10,12–14) (see Additional files 1 and 2 for example of a positive change from category score 2 to score 1 on subtest 14). A reduced muscle tone and better adjustment of the shoulder and hip muscles may be an explanation. The improvement was also pronounced on the subtests 'Passive movement of the left and right foot '(15–16), revealing problems in the calf muscles. These muscles are important in maintaining body alignment [31]. A high degree of improvement was also observed on the subtest 'Thumb movement', measuring neuromotor inhibition problems see Additional files 3 and 4 for example of a positive change from category score 2 to score 0).

It has been shown in previous studies that MPH significantly reduces activity level in children with ADHD and enhances sustained attention, as well as the speed and organization of motor response processes and motor inhibitory control [32]. Administration of MPH has also been shown to improve handwriting in children with ADHD [21,22]. A restricted movement of the shoulder, elbow and thumb may negatively affect the quality of handwriting. Our results indicate that a single dose of MPH significantly improved the movement of the thumb, the elbow (pronation-supination) and the shoulder (the subtests 'Throw ball' and 'Lifting arm') in boys with ADHD-C/HKD. It is quite possible that these improvements may account for the positive effect of MPH on handwriting shown by Tucha and Lange [21,22]. Our study also showed improved movement of the hips and feet (subtest 13–16) with MPH. Restricted hip and feet movements may negatively affect gait and jumping.

Most of the above mentioned subtests were developed to demonstrate *specific *motor problems in ADHD. For the rest of the subtests (1–4, 8, 9 and 17), which are mainly adaptations of other tests of motor function, the improvement with MPH was not so pronounced, compared to the first mentioned group of subtests. A close examination of the scoring patterns on the subtests 1–4, 8, 9 and 17 using the Change score system, revealed that the main improvement observed in these tests was rated *within *the original baseline category (+1 scores), with relatively fewer examples of change *across *categories (+2 and +3).

Our present study indicates a close correspondence between the subtest with the highest discriminative power, as shown by Stray et al[1], and the subtests showing consistently strong improvement with MPH. This finding supports our assumption that these subtests tap motor problems that are strongly related to the core neurofunctional problems of ADHD. The possible neurophysiological connection between motor problems demonstrated by the MFNU and the core problems of ADHD is discussed at length in the MFNU manual [5], and by Stray [2], and in Stray et al. [1].

What we wanted to examine in this study was the possible power of the MFNU as a predictor of MPH response in children with ADHD. The results indicate that the children with high scores on the subtests measuring problems in motor inhibition or heightened tensions in the movement- and stabilizing muscles consistently show a clear improvement rate with MPH. In our study, with a well-defined and selected HKD F-90.0 sample, 21 out of 23 subjects (91%) showed a positive field response to MPH. This result is very promising when *positive *prediction is considered. However, the study can not tell us whether children diagnosed with ADHD combined and with a *low *Total score on the MFNU will tend to show a *negative *clinical MPH response. A prior study by Stray [2], using a selection of the MFNU subtests, suggested such a relationship. This study retrospectively compared the clinical MPH results of 73 ADHD children with results of a selection of MFNU subtests obtained before diagnosis and clinical MPH evaluation of the core problems of ADHD. The results showed significantly more motor problems in positive clinical responders to MPH than in the non-responder group, indicating that a low score on the MFNU reduced the chance of positive MPH responses on attention/hyperactivity. However, more controlled studies with an ADHD sample with a higher representation of normal motor performers are needed to clarify this issue.

### Limitations

The fact that the results of the present study yield very strong support to our hypotheses makes a careful scrutiny of possible weaknesses in the design necessary. A possible weakness in the MFNU scoring system might be linked to the reliance on subjective judgements involved in deciding on the basic category scores (0–2). However, well defined scoring criteria [5], together with the documented high inter-rater reliability of the basic 3-category-scoring system makes it improbable that rater bias or other factors associated with subjective rating contributed to the results in any significant way. The perhaps most problematic part of the design is associated with the use of one scoring sheet for all observations. With repeated sessions the raters had access to prior scoring. It is quite possible that the results would have been less clear if the raters had been blinded to prior ratings by the use of separate scoring sheets for each repeated session. However, the rating procedure, which is the standard way of evaluating test-retest performance with the MFNU [5], was chosen to make an evaluation of changes in performance both *across *categories and *within *the same category possible. As illustrated by Table 7 this ability of the scoring system to detect finer differences in individual test-retest performance is particularly evident in the subtests which involve complex movements like dynamic balance and synkinesis. The use of a blinded 3-category rating of each trial would probably have missed out many of these clinically important differences. The fact that our design applying the dual screen setup and single sheet scoring has proven reliable in intertester-reliability analyses (ICC = .79 – .99), also makes it improbable that rater bias effects influenced the results of our study in any important way.

The subjects included in this study were carefully selected to identify a well-defined ADHD-C/HKD F 90.0 sample, excluding subjects with conduct disorder, depressive or anxiety disorder, Asperger or Tourette syndrome or known epilepsy or the ADHD-I or H type of clinical problems. This implies that our conclusions cannot be generalized to the broader ADHD population as defined by the DSM-IV. Other clinical conditions where co-morbid ADHD symptoms are present may show a different picture with regard to MPH response on motor problems. Since the sample consisted of boys only, and in a limited age range, reservations should also be taken when generalizing our conclusions to girls with ADHD and to older individuals.

## Conclusion

Our study demonstrated that administration of a single dose of MPH in boys diagnosed as ADHD-C/HKD F 90.0 yields a significant improvement in motor problems, as measured with the MFNU, and that this effect disappears when the MPH is metabolised. The improvements are seen in all areas assessed within the MFNU, including direct and indirect symptoms of motor disinhibition, balance, synkinesis and muscle tone of compensating muscles. The results support our earlier clinical observations and our suggestions that motor problems in ADHD may be a more integrated part of the core problems of the dysfunction than previously assumed. While a high total score on the MFNU predicts a positive MPH response on *motor performance*, further research is needed to establish the strength of the MFNU as a predictor of MPH response on the behavioural symptoms. It would also be of great interest to study ADHD subjects with little or no motor problems, both to decide if their core problems are different from ADHD subjects scoring high on MFNU, and if MPH works in a different or less effective way in these children. Further controlled studies of motor problems in ADHD in connection with MPH, may also contribute to a clarification of many of the unresolved theoretical issues associated with the neurobiological basis of ADHD.

## Competing interests

The MFNU manual is sold by the Reading Centre, University of Stavanger for NOK 700 (about €85). LLS receives 9%, TS 3% and SI 3% royalties.

## Authors' contributions

LLS has been the main contributor both in conception, design, acquisition, analysis and interpretation of data. She also is the main contributor in the drafting of the manuscript.

TS has been involved in the analysis and interpretation of data, and in the drafting and revision of the manuscript.

SI has been a contributor in the conception, design and revision of the manuscript.

AR has been a contributor to the conception, design and acquisition of data.

BE has contributed to the conception, design, interpretation of data and in revising the drafts of the manuscripts.

All authors read and approved the final manuscript

## Supplementary Material

Additional file 1 **Passive abduction – left hip ADHD no medication**. This video clip shows the MFNU subtest 'Passive abduction – left hip' performed on a child diagnosed with ADHD-C/HKD. The child had been medicated with MPH for several years. Medication was stopped one day before the video tape was made. The video clip is selected from the DVD accompanying the MFNU manual [5]. Click here for file

Additional file 2 **Passive abduction – left hip ADHD with MPH**. This video clip shows the MFNU subtest 'Passive abduction – left hip' performed on the same child as presented in the previous clip. The video tape was made 1 1/2 hr after medication with 10 mg MPH on the same day. The video clip is selected from the DVD accompanying the MFNU manual [5]. Click here for file

Additional file 3 **Thumb movement ADHD no medication**. This video clip shows the MFNU subtest 'Thumb movement' performed by the same child as presented in the previous clips. Medication was stopped one day before the video tape was made. The video clip is selected from the DVD accompanying the MFNU manual [5]. Click here for file

Additional file 4 **Thumb movement ADHD with MPH**. This video clip shows the MFNU subtest 'Thumb movement' performed by the same child as presented in the previous clips. The video tape was made 1 1/2 hr after medication with 10 mg MPH on the same day. The video clip is selected from the DVD accompanying the MFNU manual [5]. Click here for file
